# Incorporating structural plasticity into self-organization recurrent networks for sequence learning

**DOI:** 10.3389/fnins.2023.1224752

**Published:** 2023-08-01

**Authors:** Ye Yuan, Yongtong Zhu, Jiaqi Wang, Ruoshi Li, Xin Xu, Tao Fang, Hong Huo, Lihong Wan, Qingdu Li, Na Liu, Shiyan Yang

**Affiliations:** ^1^School of Health Science and Engineering, Institute of Machine Intelligence, University of Shanghai for Science and Technology, Shanghai, China; ^2^Automation of Department, Shanghai Jiao Tong University, Shanghai, China; ^3^Origin Dynamics Intelligent Robot Co., Ltd., Zhengzhou, China; ^4^Eco-Environmental Protection Institution, Shanghai Academy of Agricultural Sciences, Shanghai, China

**Keywords:** spiking neural network, self-organization, reward-modulated spike timing-dependent plasticity, homeostatic plasticity, structural plasticity

## Abstract

**Introduction:**

Spiking neural networks (SNNs), inspired by biological neural networks, have received a surge of interest due to its temporal encoding. Biological neural networks are driven by multiple plasticities, including spike timing-dependent plasticity (STDP), structural plasticity, and homeostatic plasticity, making network connection patterns and weights to change continuously during the lifecycle. However, it is unclear how these plasticities interact to shape neural networks and affect neural signal processing.

**Method:**

Here, we propose a reward-modulated self-organization recurrent network with structural plasticity (RSRN-SP) to investigate this issue. Specifically, RSRN-SP uses spikes to encode information, and incorporate multiple plasticities including reward-modulated spike timing-dependent plasticity (R-STDP), homeostatic plasticity, and structural plasticity. On the one hand, combined with homeostatic plasticity, R-STDP is presented to guide the updating of synaptic weights. On the other hand, structural plasticity is utilized to simulate the growth and pruning of synaptic connections.

**Results and discussion:**

Extensive experiments for sequential learning tasks are conducted to demonstrate the representational ability of the RSRN-SP, including counting task, motion prediction, and motion generation. Furthermore, the simulations also indicate that the characteristics arose from the RSRN-SP are consistent with biological observations.

## 1. Introduction

**Figure 1:**
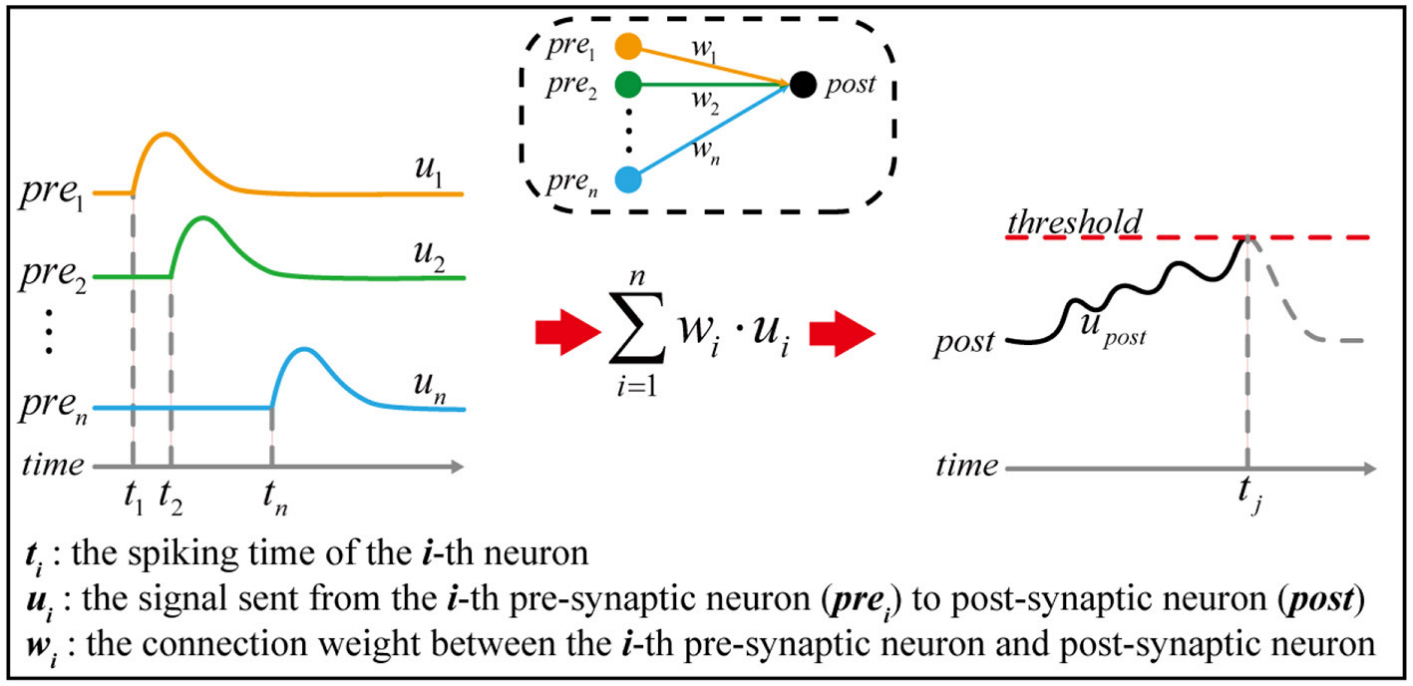
The principle of temporal encoding. Given a simple neural network in the dashed box, *n* pre-synaptic neurons are connected to one post-synaptic neuron. The pre-synaptic neuron *pre*_*i*_ generates a spike at time *t*_*i*_, which causes a signal *u*_*i*_ continuously sent to the post-synaptic neuron. Once the signal received by the post-synaptic neuron exceeds the threshold, a spike is generated, and the corresponding spiking time is marked as *t*_*j*_. According to neuroscience, information is thought to be encoded in the spiking time sequence, such as *t*_1_, *t*_2_, …, *t*_*n*_, *t*_*j*_.

Spiking neural networks, inspired by biological neural networks, are deemed to possess strong information processing abilities due to temporal encoding (as shown in Figure 1) and variable connection pattern (Zhang et al., 2018b, 2021; Bellec et al., 2020), which are driven by multiple neural plasticities, such as STDP (Frémaux and Gerstner, 2016; Brzosko et al., 2019), structural plasticity (Caroni et al., 2012; Milano et al., 2020), and homeostatic plasticity (Delvendahl and Müller, 2019; Haşegan et al., 2022). STDP enables the network to modulate its connection weight based on spike timing, while homeostatic plasticity can regulate the excitability of neurons within an appropriate range. Structural plasticity can endow the network with robust adaptability by fine-tuning its mesoscopic connection pattern during the lifecycle. However, it is non-trivial to achieve a stable training procedure for spiking neural networks incorporating multiple neural plasticity mechanisms. The temporal encoding of biological neural networks is a sophisticated information encoding method, which needs to cooperate with various neural plasticities to exert strong information processing ability. Although many sophisticated spiking neuron models have been designed, there is a lack of research on neural plasticities. Many current spiking neural networks are simple abstraction of biological neural networks, which makes it not an easy task to train the network. For example, changes in input may occasionally cause a sharp increase or decrease in the firing rate of neurons, resulting in probabilistic non-convergence (Pfeiffer and Pfeil, 2018; Xing et al., 2019) and a lack of adaptation to input (Wang et al., 2020). To address these questions, it is crucial to understand how these plasticities interact to shape neural networks and affect neural signal processing. But, it is difficult and expensive to directly observe the biological neural network at the mesoscopic level, since it consists of a large number of neurons that are dynamically connected through synapses.

To handle the problem mentioned above, it is a potential way to establish a biologically reasonable spiking neural network model that incorporates multiple neural plasticity mechanisms. For example, Lazar et al. proposed a self-organization recurrent network (SORN) driven by multiple neural plasticities (Lazar et al., 2009), which only consists of a recurrent layer and an output layer. The recurrent layer is mainly adapted by STDP and homeostatic plasticity. STDP is used to adjust synaptic weights based on postsynaptic spike activity. In detail, synaptic weight is strengthened when pre-synaptic spike activity is followed by post-synaptic spike activity, while the reverse pattern makes synaptic weight weak. Homeostatic plasticity induces a competition among synaptic connections and maintains spike firing. The simulation results of SORN show that STDP and homeostatic plasticity lead to some non-statistical characteristics of spiking neural networks, such as lognormal-like distribution of synaptic weights, long-term persistence of strong synaptic connections, and power-law distribution of synaptic lifetimes. Inspired by SORN, Aswolinskiy et al. proposed a reward-modulated self-organization recurrent network (RM-SORN) (Aswolinskiy and Pipa, 2015; Dora et al., 2016), in which synaptic weights are adjusted by R-STDP and homeostatic plasticity. R-STDP refers that the outcome of STDP, induced by pre-synaptic and post-synaptic spike activity, is gated by external reward, and the resulting learning rules are no longer unsupervised (Izhikevich, 2007; Anwar et al., 2022).

Despite the fact that the SORN and RM-SORN models are self-organization networks, their connection patterns do not alter continually during the training phase. It means that these spiking neural network models do not really incorporate structural plasticity, and structural plasticity remains under-explored for existing SNNs. Therefore, in this work, we propose a novel reward-modulated self-organization recurrent network with structural plasticity, in which the connection pattern is continuously adjusted along with the lifecycle. In detail, R-STDP is utilized to generate effective representations for inputs in the recurrent layer, which also helps to achieve efficient mapping in the output layer. Besides, homeostatic plasticity is used to stabilize the excitability of neurons. In particular, structural plasticity is further introduced to simulate the growth and pruning of connections in the recurrent layer, which could well explore the characteristics of structural plasticity for training SNNs. The representational ability of the RSRN-SP is evaluated on three sequence learning tasks, including counting task, motion prediction task, and motion generation task.

In summary, our contributions are as follows: (1) We propose a novel reward-modulated self-organization recurrent network with structural plasticity (RSRN-SP), in which structural plasticity is introduced from neurophysiology to enhance the variability of connection patterns; (2) We experimentally find that structural plasticity could improve the adaptability of the network and reduce the training difficulty; (3) We empirically reveal some characteristics arose from the RSRN-SP are consistent with biological observations, i.e., lognormal-like distribution of connection weight, power-law distribution of connection lifecycle, and a stable tendency for stronger connections; (4) Experiments on three sequence learning tasks show that our method achieve better representation ability than the same type of spiking neural networks such as SORN and RM-SORN. Further analyzes are utilized to demonstrate the effectiveness of structural plasticity.

## 2. Related works

There have been many researches on spiking neuron models and learning rules (Yu et al., 2014; Zhang et al., 2018a; Ju et al., 2020; Xu et al., 2021), where neuron models, learning rules, and network architectures are three essential factors for designing spiking neural networks.

### 2.1. Neuron models

The human brain contains billions of neurons, which form structurally complex and computationally efficient networks through dynamic synaptic connections (Bassett and Sporns, 2017). There are various spiking neural models to simulate the temporal coding of neurons in the brain, i.e., Hodgkin-Huxley (HH) model (Izhikevich, 2004), leaky integrate-and-fire (LIF) model (Yu et al., 2014), and binary neuron model (Dayan and Abbott, 2001). The HH model focuses on the microscopic mechanism of spikes, while the LIF model focuses on the computational complexity of spikes. The binary neuron model is developed from the LIF model, has lower computational complexity, and is suitable for building large-scale networks. Therefore, in this work, the binary neuron model is used to construct a spiking neural network.

### 2.2. Learning rules

Inspired by biological observations, there are mainly two brain-inspired learning rules suitable for SNNs (Caporale and Dan, 2008; Frémaux and Gerstner, 2016): Hebb learning rule and STDP learning rule. The former suggests that neurons activated at the same time should have a closer relationship. The latter indicates that synaptic weight is adjusted based on the spike timing of pre-synaptic and post-synaptic neurons. STDP learning rule can be described as follows,


(1) $$\Delta w_{ij}=\{\begin{array}{l} A_{+}\exp\left(-\Delta t/\tau_{+}\right),\text{ if }\Delta t\geq 0\\ -A_{-}\exp\left(\Delta t/\tau_{-}\right),\text{ if }\Delta t<0 \end{array}$$

where Δ*w*_*ij*_ represents the weight change of connection from pre-synaptic neuron *j* to post-synaptic neuron *i*. *A*_+_, *A*_−_, τ_+_, and τ_−_ are dimensionless constants, which are obtained by fitting neurophysiological data. $\Delta t=t_{i}^{f}-t_{j}^{f}$ represents the error between the last spike timing of post-synaptic neuron *i* and the last spike timing of pre-synaptic neuron *j*.

### 2.3. Spiking neural networks

Spiking neural networks are known as the third-generation neural networks. Due to the brain-like temporal encoding and multiple neural mechanisms, they are considered to have a strong information processing capability. However, this also makes the training of spiking neural networks difficult (Wang et al., 2020). The widely used gradient descent algorithm is difficult to apply to spiking neural networks (Taherkhani et al., 2020). Therefore, many researches combine various mathematical optimization techniques with spiking neural networks, trying to propose a new learning paradigm suitable for SNNs. For example, Xing et al. adopted the ANN-to-SNN strategy to migrate the parameters of the trained ANNs to the SNNs of the same architecture (Xing et al., 2019; Gao et al., 2023). Anwar et al. applied reinforcement learning to spiking neural networks to perform specific tasks, such as Pong and Cartpole game playing (Bellec et al., 2020; Anwar et al., 2022; Haşegan et al., 2022). These spiking neural networks combine biologically reasonable neural mechanisms with reinforcement learning and mathematical optimization to complete sophisticated tasks. Some studies also employ spiking neural networks containing biologically reasonable neural mechanisms to explore the principle of biological neural network information processing. For example, the SORN and RM-SORN models are proposed to explore the coordination of neural mechanisms such as R-STDP, structural plasticity, and homeostatic plasticity (Lazar et al., 2009; Aswolinskiy and Pipa, 2015; Dora et al., 2016).

## 3. The proposed method

### 3.1. Problem formulation

#### 3.1.1. Preliminary

Consider a binary neuron model, the neuron state (0 and 1) changes over the inputs. The binary neuron state *s*_*t*_ at discrete time *t* is updated as follows:


(2) $$s_{t}=\{\begin{array}{l} 1,\text{ if }\psi_{t}\geq \theta \\ 0,\text{ if }\psi_{t}<\theta \end{array}$$

where θ is the threshold and ψ is the sum of inputs. Once ψ reaches the threshold, the neuron state will be activated as 1, otherwise *x* = 0. Besides, ψ is calculated as follows:


(3) $$\begin{array}{l} \psi_{i}=u_{i}+\underset{j\in S}{\sum }w_{ij}s_{i} \end{array}$$

where *w*_*ij*_ is the weight between neuron *i* and neuron *j*. *u*_*i*_ is the external signal received by neuron *i*. The notations used in this work are explained in Table 1.

**Table 1:** Notations used in this work. We demonstrate the commonly used notations in this work.

**Notation**	**Interpretation**
*I*/*E*	Inhibitory/Excitatory neuron
*N*^*I*^/*N*^*E*^	Number of inhibitory/excitatory neuron
$s_{i}^{e}/s_{i}^{in}/s_{i}^{o}$	State of *i*-th excitatory/inhibitory/output neuron
$\theta_{i}^{e}/\theta_{i}^{in}/\theta_{i}^{o}$	Threshold of *i*-th excitatory/inhibitory/output neuron
$w_{ij}^{ee}$	Weight between *i*-th excitatory and *j*-th excitatory neuron
$w_{ij}^{ei}$	Weight between *i*-th excitatory and *j*-th inhibitory neuron
$w_{ij}^{ie}$	Weight between *i*-th inhibitory and *j*-th excitatory neuron
$w_{ij}^{oe}$	Weight between *i*-th output and *j*-th excitatory neuron

#### 3.1.2. Counting task

The task objective is to predict the subsequent element by modeling the structure of a recurrent sequence. Consider a *m* times recurrent sequence as follows:


(4) $$\begin{array}{l} \overset{1}{\overset{︷}{a\underset{n}{\underset{︸}{bb\cdots b}}c}}\overset{2}{\overset{︷}{a\underset{n}{\underset{︸}{bb\cdots b}}c}}\cdots \overset{m}{\overset{︷}{a\underset{n}{\underset{︸}{bb\cdots b}}c}} \end{array}$$

where each subsequence contains a start flag, an end flag and a fixed number of a repeated element (i.e., *a*, *c*, and *b*). Taking an element as the input, the model aims to accurately predict the next element. For example, given *a* as the input, and the ground-truth is *b*. This task is designed to test the memory property of the model.

#### 3.1.3. Motion prediction task

The task objective is to predict the subsequent element by modeling the structure of a recurrent sequence, in which all elements are associated with different spatial positions. Consider a *m* times recurrent sequence as follows:


(5) $$\begin{array}{l} \overset{1}{\overset{︷}{12\cdots n}}\overset{2}{\overset{︷}{12\cdots n}}\cdots \overset{m}{\overset{︷}{12\cdots n}} \end{array}$$

where each subsequence contains *n* integers from 1 to *n*. Taking an element as the input, the model aims to accurately predict the next element. Because the elements are associated with different spatial positions, this task can be interpreted as the left to right motion of an object along an axis.

#### 3.1.4. Motion generation task

The task objective is to generate the *m* times recurrent sequence same as (5), with the output serving as the input and no external teaching signals. For example, if the current output of the model is equal to 1 and serves as the next input, the next output should be 2.

### 3.2. Overview architecture

**Figure 2:**
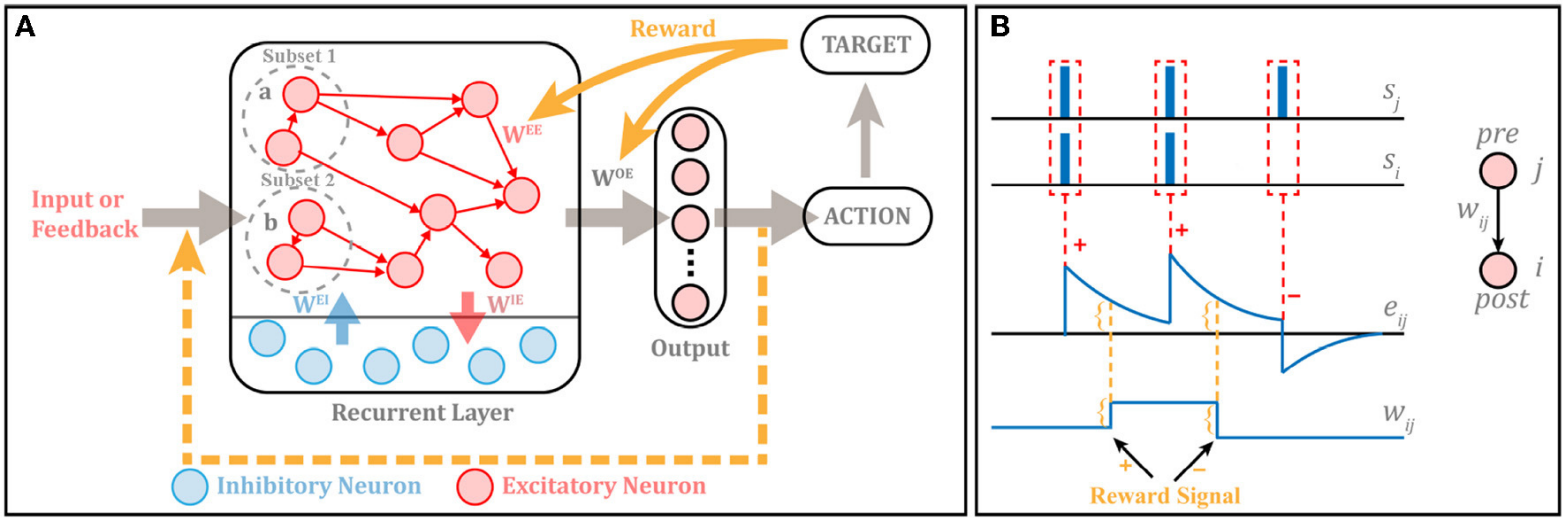
**(A)** The structure of RSRN-SP. Excitatory-to-Excitatory (E-E) connections *W*^*EE*^ are sparse. Excitatory-to-Inhibitory (E-I) connections *W*^*IE*^, Inhibitory-to-Excitatory (I-E) connections *W*^*EI*^ and connections from the recurrent to output layer *W*^*OE*^ are full. *W*^*EE*^ and *W*^*OE*^ follow R-STDP, while others keep fixed. Suppose that the input is a *m* times recurrent sequence (i.e., *abab*...*ab*), in which *a* and *b* correspond to subset 1 and 2, respectively. When *a* is input into the model, only the neurons of the subset 1 are activated, likewise *b* is input into the model, only the neurons of the subset 2 are activated. **(B)** Illustration of R-STDP. The outcome of R-STDP is stored in eligibility trace. The updating of the weights occurs only at the time of reward.

The overall architecture of RSRN-SP is shown as in Figure 2, which is composed of two general modules, i.e., a recurrent layer, and an output layer.

#### 3.2.1. Recurrent layer

The recurrent layer extracts features of inputs and stores them in its variable connection patterns and weights. The neurons in the layer are divided into two groups: excitatory and inhibitory neurons. *N*^*E*^ and *N*^*I*^ are utilized to denote the numbers of them, *N*^*I*^ = 0.2 × *N*^*E*^. They are connected through weighted connections, which is denoted as a weight matrix *W*. The element *w*_*ij*_ in the weight matrix is the connection weight from neuron *j* to neuron *i*. The connections among excitatory neurons are sparse, while full connections exist between excitatory and inhibitory neurons. The initial connection density among excitatory neurons is controlled by the average connection fraction *p*_*c*_ of neurons. It should be noted that there exist no self-connections and connections among inhibitory neurons.

#### 3.2.2. Output layer

The output layer maps the features stored in the recurrent layer into interpretable and specific-task outputs. It only contains excitatory neurons without connections among each other. There is a feedback connection from the output layer to the recurrent layer.

### 3.3. Evolution

The input of RSRN-SP is a time sequence that contains different symbols (letters or digits). Each symbol corresponds to a subset of neurons in the recurrent layer, which and only which can receive the corresponding input symbol. When a certain symbol in the sequence is input to the model, only neurons in the corresponding subset are activated, while other neurons remain silent. The state updating for different types of neurons are as follows:


(6) $$\begin{array}{l} s_{k}^{e}(t+1)\\ =\Theta (\sum_{i=1}^{N^{E}}w_{ki}^{ee}s_{i}^{e}(t)-\sum_{j=1}^{N^{I}}w_{kj}^{ei}y_{k}(t)+u_{k}(t)-\theta_{k}^{e}(t)), \end{array}$$

(7) $$s_{k}^{in}(t+1)=\Theta (\sum_{j}^{N^{E}}w_{kj}^{ie}s_{j}^{e}(t)-\theta_{k}^{in}(t)).$$

(8) $$\begin{array}{l} s_{k}^{o}\left(t+1\right)=\Theta \left(\overset{N^{E}}{\underset{j}{\sum }}w_{kj}^{oe}s_{j}^{e}\left(t\right)-\theta_{k}^{o}\left(t\right)\right) \end{array}$$

where Θ is the Heaviside step function, and *u*_*k*_(*t*) is the external signal of neuron *k* at time *t*. The weights are uniformly drawn from [0, 1] and initially normalized as $\overset{N}{\underset{j}{\sum }}w_{ij}=1$.

### 3.4. Learning rule of R-STDP

According to the learning rule of R-STDP (Frémaux and Gerstner, 2016; Yuan et al., 2018), the change of connection weight is not only controlled by spikes, but also regulated by reward signal, as illustrated in Figure 2B, which can be described as follows:


(9) $$\begin{array}{l} W\left(t+1\right)=W\left(t\right)+\eta \cdot M\times e. \end{array}$$

Where η is the learning rate of the weight. *e* denotes a synaptic eligibility trace to temporarily store the outcome of R-STDP, which can be still available for a delayed reward signal. The eligibility trace is computed as:


(10) $$\begin{array}{l} \frac{de_{ij}}{dt}=-\frac{e_{ij}}{\tau_{e}}+[s_{i}(t)\cdot s_{j}(t-1)\\ \;-f\cdot s_{i}(t-1)\cdot s_{j}(t)] \end{array}$$

where *s*_*i*_ and *s*_*j*_ are the activation of pre- and post-synaptic neurons, respectively. τ_*e*_ is a time constant. *f* is a dimensionless parameter (*f* = 1 for *W*^*EE*^ and *f* = 0.01 for *W*^*OE*^). According to Equation (9), when neuromodulation factor *M* = *R*−*b* is not equal to 0, i.e., reward *R* deviates from baseline *b*, the connection weight will be updated.

In our model, for the counting task and motion prediction task, the reward *R* is set to 1 for correct output, while either 0 or −1 for incorrect output. The baseline *b* is set to the moving average of *R*. For the generation task, when the target sequence is correctly generated, the reward *R* that is proportional to the length of the correctly generated sequence will be given.

### 3.5. Structural plasticity

Structural plasticity is a fundamental neural mechanism of the biological neural network in the brain, which is demonstrated to have a critical role in regulating circuit connection during learning (Caroni et al., 2012). Structural plasticity refers that old synaptic connections may be pruned and new synaptic connections formed during the self-organization of neural networks (Lamprecht and LeDoux, 2004). In our model, we apply structural plasticity to the connections among excitatory neurons. New connections will be added between two unconnected neurons with a probability *p*_*sp*_∈(0, 1), and their weights are initialized as 0.001. *p*_*sp*_ is fine-tuned as a hyper-parameter to stabilize the recurrent layer. Old connections will be pruned if their weights are less than a near-zero threshold *w*^*th*^∈(0, 1).

Structural plasticity can be formulated as follows:


(11) $$\{\begin{array}{l} c_{ij}=0,\text{ if }c_{ij}=1\text{ and }w_{ij}<w^{th}\\ c_{ij}=1,w_{ij}=0.001,\text{ if }c_{ij}=0\text{ and }rnd>1-p_{sp} \end{array}$$

where *c*_*ij*_∈{0, 1} indicates whether a connection from neuron *j* to neuron *i* exists or not. If *c*_*ij*_ = 1, the connection exists; if *c*_*ij*_ = 0, the connection does not exist. *rnd*∈(0, 1) is a uniformly distributed random number. *w*_*ij*_ denotes the weight of connection from neuron *j* to neuron *i*.

### 3.6. Homeostatic plasticity

Homeostatic plasticity is critical to alleviate the instability of neural networks. Two common homeostatic mechanisms are utilized in our method: synaptic normalization and intrinsic plasticity. The synaptic normalization is formulated as:


(12) $$\begin{array}{l} w_{ij}\left(t\right)=\frac{w_{ij}\left(t\right)}{\underset{j}{\sum }w_{ij}\left(t\right)}\to \underset{j}{\sum }w_{ij}\left(t\right)=1. \end{array}$$

Where the weights of all afferent connections to a neuron are proportionally scaled to make their sum equal to 1. Synaptic normalization can promote healthy competition among connections that connect to the same neuron.

The intrinsic plasticity enables to adjust the thresholds of excitatory neurons by an average firing rate μ_*ip*_, which can be formulated as follows:


(13) $$\begin{array}{l} \Delta \theta_{i}^{e}\left(t\right)=\eta \left(s_{i}\left(t\right)-\mu_{ip}\right). \end{array}$$

Where η_*ip*_ is the learning rate of the threshold. For excitatory neurons in the recurrent layer, μ_*ip*_ is fine-tuned in a range of [0.05, 0.25]. In the output layer, μ_*ip*_ is uniquely set for each neuron, corresponding to the expected occurrence probability of the symbol represented by the neuron. Due to the intrinsic plasticity, the threshold of a neuron in our model will increase if it is too active; otherwise, the threshold will decrease.

### 3.7. Two-stage training

A two-stage training scheme is proposed for RSRN-SP. In the first stage, the model is trained with R-STDP, homeostatic plasticity, and structural plasticity. In the second stage, the connection pattern and weight of the recurrent layer are fixed. The connection weight of the output layer is fine-tuned for specific tasks. The first stage is alternated with the second stage. In each alternation, the model takes about 100 steps at the first stage, and then proceeds to the second stage to take about 20,000 steps. During training, the alternation will be repeated about 200 times. During inference, the model will be evaluated on testing data. For the motion generation task, the output of the model is fed back as its input during training, and the model is used to generate desired output during inference. The algorithm for the first stage is listed here. The second-stage algorithm is similar, which only applies R-STDP and homeostatic plasticity at the output layer.

**Algorithm 1:**
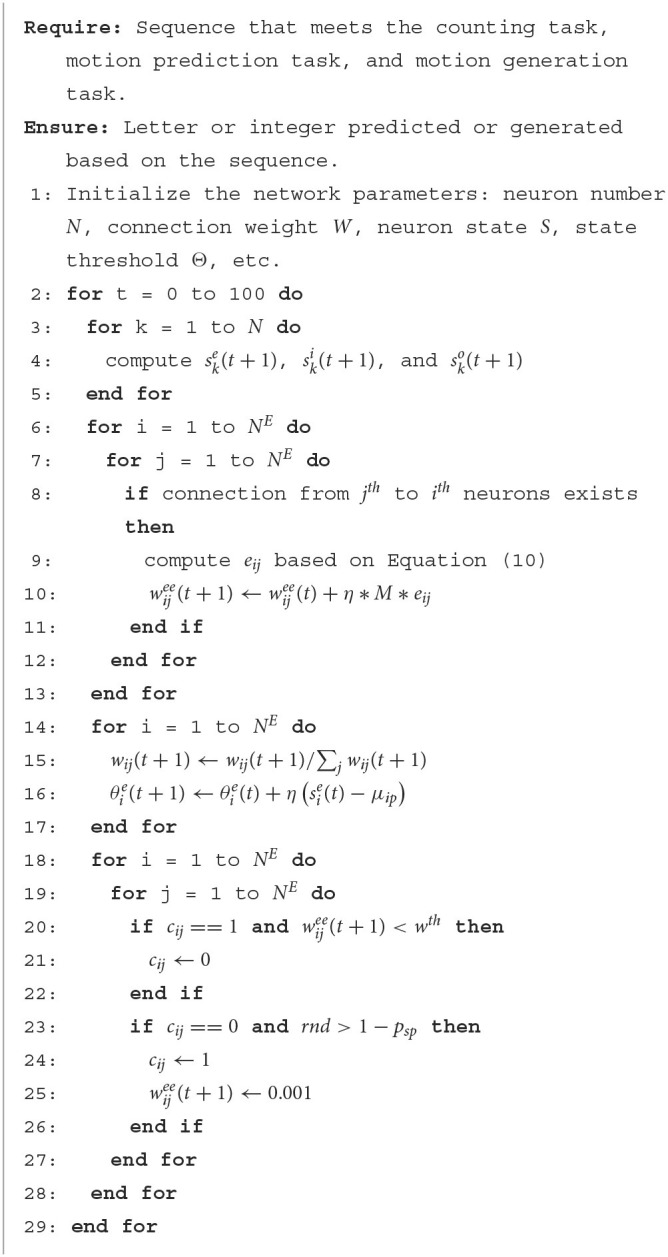
The first-stage algorithm of RSRN-SP.

## 4. Experiments

### 4.1. Experimental settings

Our model is evaluated on three tasks: counting task (Lazar et al., 2009), motion prediction (Aswolinskiy and Pipa, 2015), and motion generation (Aswolinskiy and Pipa, 2015). The comparison approaches mainly involve SORN (Lazar et al., 2009) and RM-SORN (Aswolinskiy and Pipa, 2015). Notably, in SORN and RM-SORN, the weights to the output layer are trained with linear regression, and there is no structural plasticity applied in the recurrent layer. The model is implemented in Python programming on a Windows 10 computer with NVIDIA RTX 1080Ti. Source code and parameters are released at Github.

### 4.2. Experiments for counting task

#### 4.2.1. Evaluation protocol

Two kinds of evaluation protocols are used: (1) overall performance is to evaluate the matching of all letters in a sequence; (2) counting performance is to evaluate the prediction accuracy for all subsequences in a sequence.

#### 4.2.2. Results

**Figure 3:**
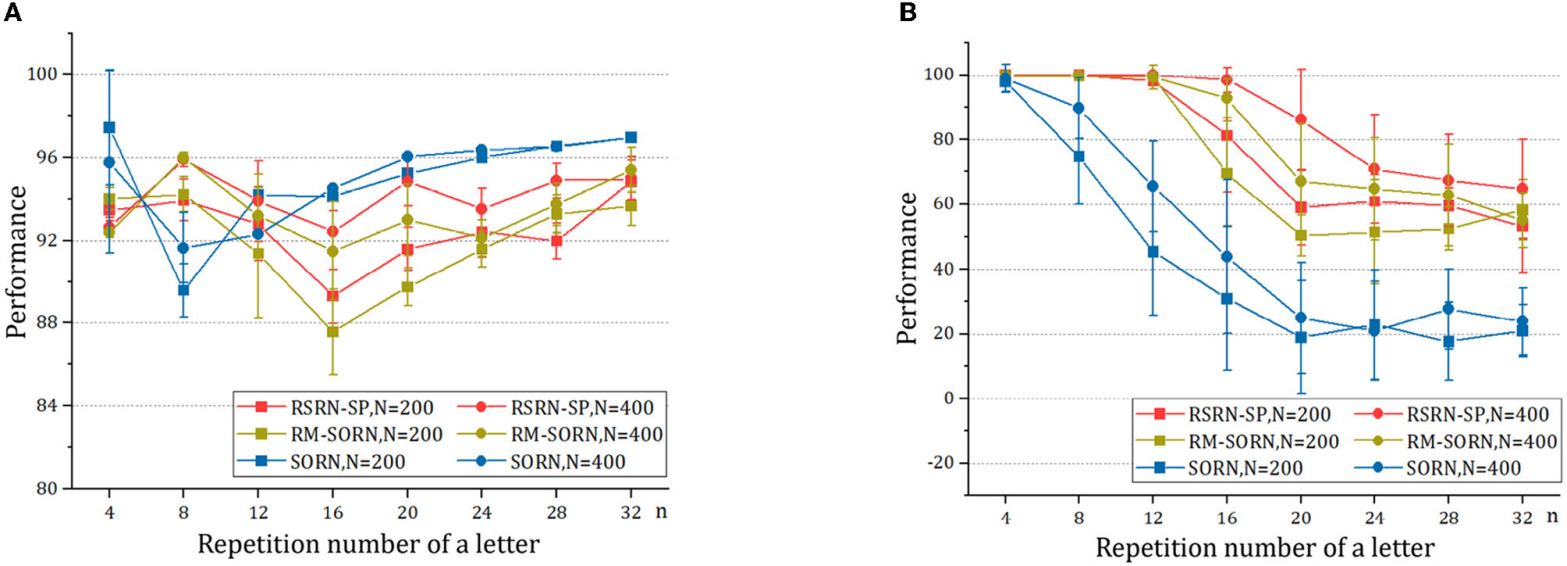
The results of the counting task. **(A)** The overall performance. **(B)** The counting performance.

The results for the counting task are shown in Figure 3 and Table 2. It can be observed that as the number *n* of the repetition of a letter in a subsequence increases, the overall performance fluctuates around 90% within a relatively narrow range, while the counting performance declines. This is because the value of *n* is proportional to the number of input patterns that the recurrent layer needs to learn. Larger *n* increases the difficulty of predicting the last letter of a subsequence, but reduces the difficulty of predicting the other letters of this subsequence. The counting performance gap among our model, SORN, and RM-SORN can be explained by the difference between reward-modulated learning and offline linear regression. SORN and RM-SORN try to learn all separable input patterns and minimize global mapping errors, whereas the reward of our model is computed as moving average within a time window, in which each individual input pattern is more effectively learned.

**Table 2:** The results of the counting task.

**Overall performance**	***n***				
*N* = 200	4	8	12	16	20
RM-SORN	94.02	94.23	91.39	87.56	89.73
RSRN-SP (Ours)	93.48	93.96	**92.82**	**89.28**	**91.59**
*N* = 400	4	8	12	16	20
SORN	95.78	91.66	92.31	94.52	96.04
RSRN-SP (Ours)	92.64	**95.92**	**93.91**	92.43	94.58
**Counting performance**	*n*				
*N* = 200	4	8	12	16	20
RM-SORN	99.70	99.50	99.31	69.50	50.44
RSRN-SP (Ours)	**100.00**	**100.00**	**98.18**	**81.17**	**58.88**
*N* = 400	4	8	12	16	20
SORN	98.87	89.68	65.44	43.89	24.95
RSRN-SP (Ours)	**100.00**	**100.00**	**100.00**	**98.38**	**86.04**

The bold values indicate that the method outperforms other models under the same conditions.

### 4.3. Experiments for motion prediction task

#### 4.3.1. Results

**Figure 4:**
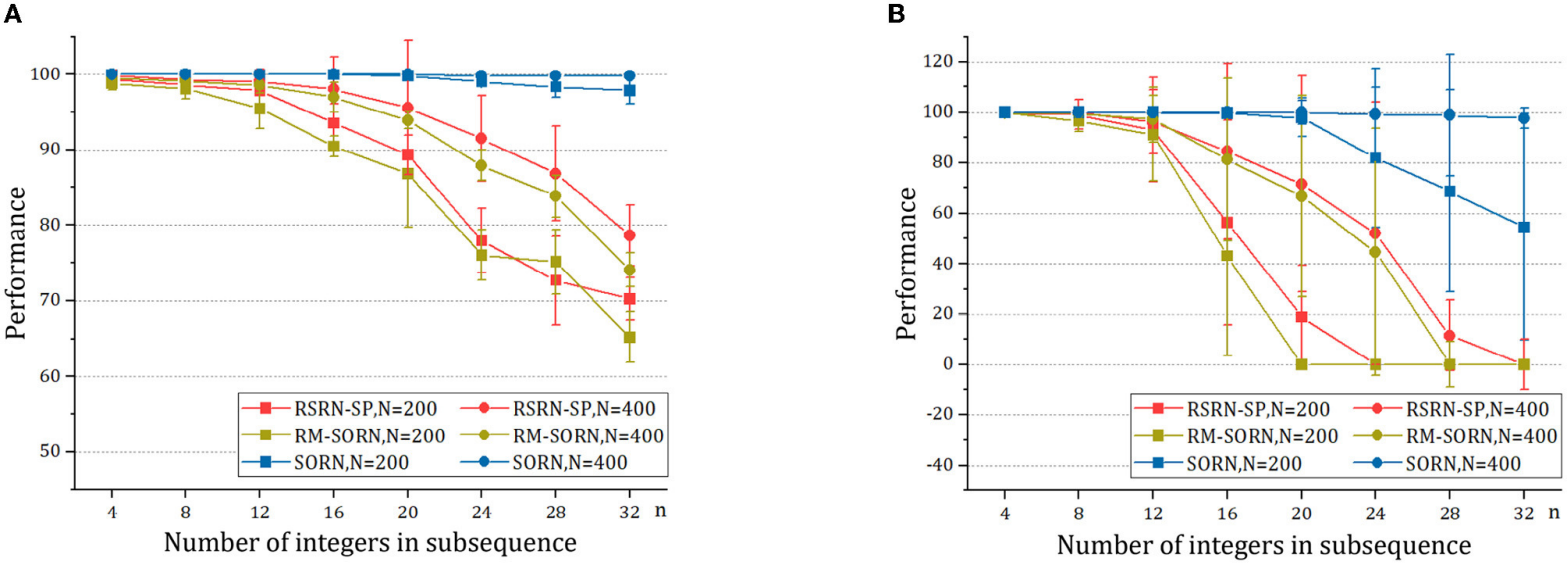
The results of the motion prediction and generation tasks. **(A)** The motion prediction task. **(B)** The motion generation task.

As shown in Figure 4A and Table 3, when *n* is small, our model, SORN and RM-SORN have high prediction accuracy. As *n* increases, the accuracy of the three models gradually decreases, and the accuracy of our model becomes higher than RM-SORN and lower than SORN. It is worth noting that in our model, increasing the number of neurons can greatly increase the performance of the model. Considering that our model has much less training difficulty than SORN, it can achieve an accuracy similar to SORN by increasing the number of neurons when *n* is very big.

**Table 3:** The results of the motion prediction task.

***N* = 200**	***n***				
	4	8	12	16	20
RM-SORN	98.79	98.04	95.42	90.46	86.80
RSRN-SP (Ours)	**99.38**	**98.63**	**97.87**	**93.51**	**89.32**
*N* = 400	4	8	12	16	20
RM-SORN	99.46	99.08	98.57	96.96	93.92
RSRN-SP (Ours)	**99.83**	**99.28**	**99.07**	**97.99**	**95.57**

The bold values indicate that the method outperforms other models under the same conditions.

### 4.4. Experiments for motion generation task

#### 4.4.1. Evaluation protocol

The performance is calculated as the percentage of the symbols belonging to the target sequence to the total number of symbols. For example, assuming that the desired sequence is 1234. If the desired sequence is generated, the model receives the full reward of unit 1. Otherwise, it receives the reward of $\frac{3}{4}$ for the sequence *x*123, $\frac{2}{4}$ for the sequence *xx*12 and $\frac{1}{4}$ for the sequence *xxx*1.

#### 4.4.2. Results

As shown in Figure 4B and Table 4, without external teaching signals, our model can still generate the desired sequence accurately. Success in this task shows that the model can generate an arbitrary sequence with the same symbol distribution as in the motion sequence.

**Table 4:** The results of the motion generation task.

***N* = 200**	***n***				
	4	8	12	16	20
RM-SORN	99.79	96.66	91.24	43.20	0.00
RSRN-SP (Ours)	**99.90**	**98.98**	**93.13**	**56.40**	**18.78**
*N* = 400	4	8	12	16	20
RM-SORN	99.92	99.66	97.24	81.20	66.70
RSRN-SP (Ours)	**100.00**	**99.98**	**96.13**	**84.64**	**71.54**

The bold values indicate that the method outperforms other models under the same conditions.

As a result of the self-organization driven by multiple plasticity, the recurrent layer can an create effective representation of inputs. The model containing 400 neurons outperforms that containing 200 neurons, suggesting that the memory capacity of the model is closely related to the number of neurons.

### 4.5. Influence of structural plasticity

**Figure 5:**
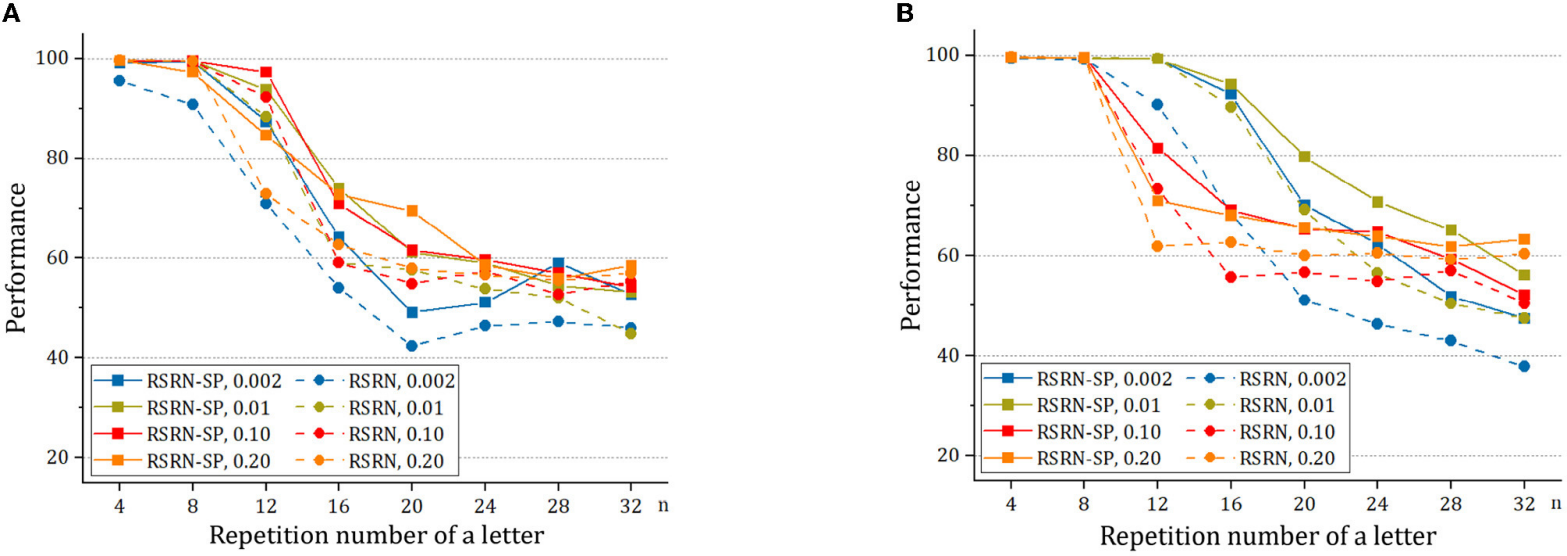
The performance of RSRN with and without structural plasticity (SP), with initial *p*_*c*_ = 0.002, 0.01, 0.1, 0.2, on the counting task. **(A)** The network consisting of *N* = 200 neurons. **(B)** The network consisting of *N* = 400 neurons.

To study structural plasticity, the models with different *N* and *p*_*c*_ are constructed in the counting task. Figure 5 and Table 5 suggest that the recurrent layer with structural plasticity outperforms that without structural plasticity. The performance improvement is larger when the recurrent layer is initialized with sparse connectivity. The recurrent layer with structural plasticity has great advantage, which is prominent when the connection patterns are not reasonably initialized. In the case of initial *p*_*c*_ = 0.002, the connection fractions change into 0.011 and 0.008 after training, for the model of *N* = 200 and *N* = 400, respectively. When the initial *p*_*c*_ is set to 0.01, the increment is much smaller. The decrease is much smaller for *p*_*c*_ = 0.1, compared to the case of *p*_*c*_ = 0.2. It is testified that the model of *N* = 200 and *N* = 400 achieved the best performance with *p*_*c*_ = 0.05 and *p*_*c*_ = 0.0125, respectively.

**Table 5:** The changes of E-E connection fractions of RSRN-SP on the counting tasks at four initial connection fractions.

** ${p_{c}}^{\left(0\right)}$ **	***N*** = 200		***N*** = 400	
	$\Delta p_{c}/{p_{c}}^{\left(0\right)}$	${p_{c}}^{\left(1\right)}$	$\Delta p_{c}/{p_{c}}^{\left(0\right)}$	${p_{c}}^{\left(1\right)}$
0.002	+(459 ± 242)%	0.011	+(305 ± 223)%	0.008
0.01	+(53 ± 29)%	0.015	+(53 ± 20)%	0.015
0.1	−(37 ± 11)%	0.063	−(41 ± 13)%	0.059
0.2	−(46 ± 10)%	0.108	−(50 ± 11)%	0.099

${p_{c}}^{\left(0\right)}$ is the initial connection fraction.

${p_{c}}^{\left(1\right)}$ is the connection fraction after the training, which is computed as the average of the model on the counting tasks (*n* = [4, 8, .., 32]).

The relative change of $\Delta p_{c}/{p_{c}}^{\left(0\right)}$ is denoted as “(mean ± std)”.

### 4.6. Synaptic connection characteristics of RSRN-SP

**Figure 6:**
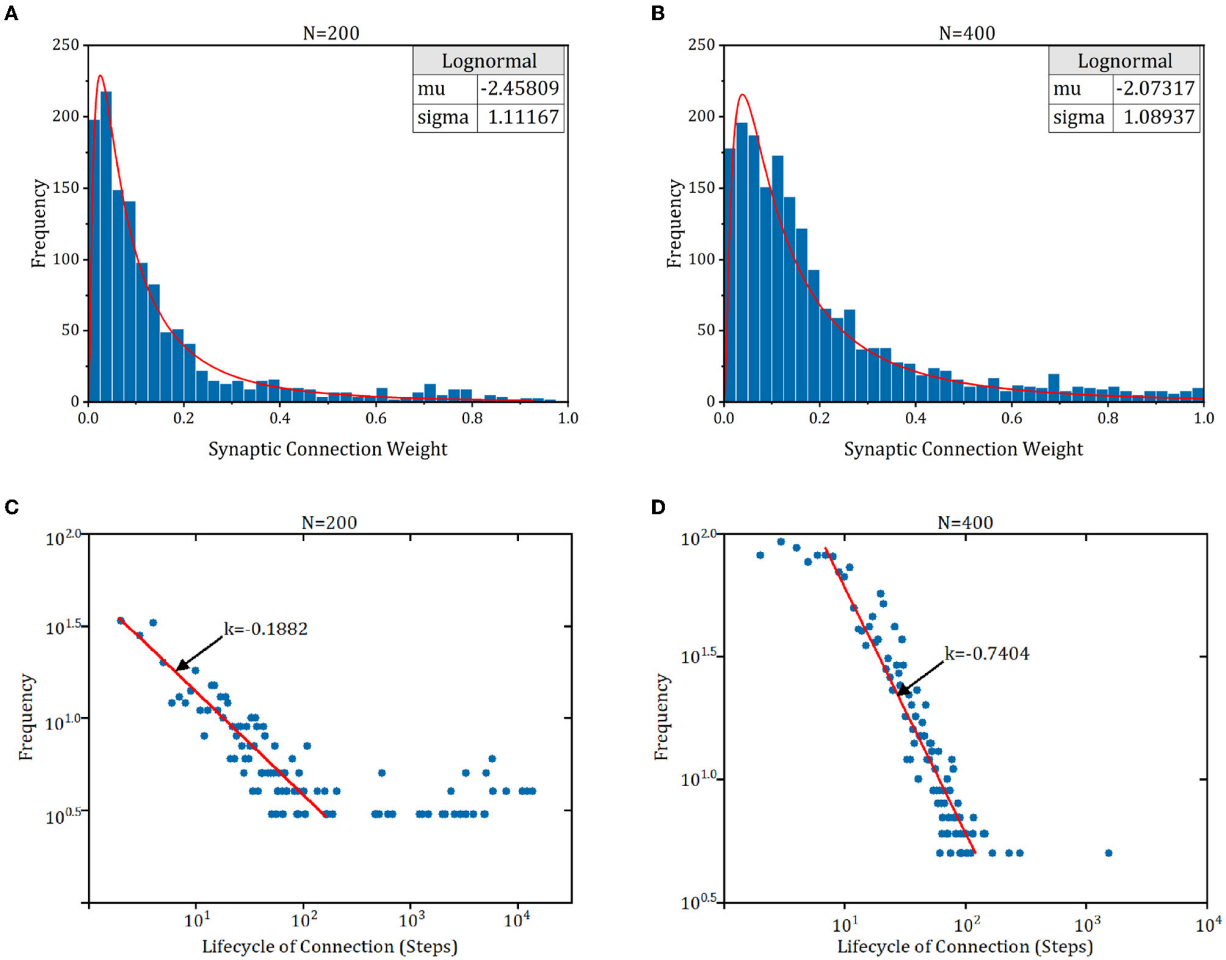
The synaptic connection characteristics of RSRN-SP. **(A, B)** Synaptic weight distribution. Blue bars are synaptic weight distribution histogram. Red line is lognormal distribution fit curve. **(C, D)** Synaptic lifecycle distribution. Red line is distribution fit curve.

The connection pattern of cortex exhibits some fundamental characteristics (Zheng et al., 2013), e.g., lognormal-like distribution of synaptic weight, power-law distribution of synaptic lifecycle, and a tendency for stronger connections to be more stable. To study whether these characteristics exist in RSRN-SP, we simulated a model of 200 and 400 on the counting task of *n* = 8. As shown in Figures 6A, B, the synaptic weights exhibit lognormal-like distribution, which is consistent with biological observations (Song et al., 2005; Loewenstein et al., 2011). Figures 6C, D demonstrated that the distribution of lifecycle of newly formed connections can be roughly fitted by a power law. Most of newly formed connections tend to disappear and only a few of them can persist and become strong.

## 5. Discussion and conclusion

To understand how multiple plasticities interact to shape biological neural networks and affect neural signal processing, we proposed a novel spiking neural network incorporating with multiple neural plasticity from neurophysiology, e.g., reward-modulated spike timing-dependent plasticity, homeostatic plasticity, and structural plasticity. In particular, homeostatic plasticity and reward-modulated spike timing-dependent plasticity are used to promote the consistency between the network updating and brain learning, which help to guide the updating of connection weight during training SNNs. Specially, structural plasticity is introduced to simulate the growth and pruning of connections in the network, which could guarantee the consistency between the network structure and brain structure.

Here, our work attempts to combine R-STDP with other plasticity mechanisms to achieve better training results. The simulations demonstrated that (1) reward-modulated spike timing-dependent plasticity, structural plasticity, and homeostatic plasticity can work in coordination to empower neural networks to learn; (2) structural plasticity weakens the network connection stability but enhances its ability to adapt to the input; (3) RSRN-SP could effectively learn the representation of the input, and achieves better performance on sequence learning tasks than the same type of spiking neural network including SORN and RM-SORN. Furthermore, the simulations also indicate that the characteristics arose from RSRN-SP are consistent with biological observations.

Compared to the widely used artificial neural networks, our spiking neural network is not easy to train due to complex temporal encoding, variable connection pattern, and diverse plasticity mechanisms. One challenge stems from the temporal encoding, which allows information to be processed in the form of spikes in SNNs. However, spikes are not mathematically differentiable, making it difficult to apply traditional gradient-based optimization algorithms. The generation of new connections and the disappearance of old connections also increase the difficulty of network training. To address these challenges, some studies have explored R-STDP (Frémaux and Gerstner, 2016), which is considered a biologically plausible learning algorithm suitable for SNNs. Nevertheless, how efficient learning of SNNs can be achieved by R-STDP, while maintaining sustained balanced network activity remains an open question.

## Data availability statement

The original contributions presented in the study are included in the article/supplementary material, further inquiries can be directed to the corresponding author.

## Author contributions

YY: software, formal analysis, and writing—original draft. RL, TF, QL, and HH: writing—reviewing and editing. XX and LW: data curation. NL: supervision and funding acquisition. All authors contributed to the article and approved the submitted version.
